# The Real-Time Support Role of Augmented Reality Technology in Shared Decision-Making in Neurosurgery Under the SEGUE Framework: Randomized Controlled Trial

**DOI:** 10.2196/87198

**Published:** 2026-04-17

**Authors:** Zhengbo Yuan, Zhongjie Shi, Zhanxiang Wang

**Affiliations:** 1 Department of Neurosurgery The First Affiliated Hospital of Xiamen University School of Medicine, Xiamen University Xiamen, Fujian China; 2 National Institute for Data Science in Health and Medicine Xiamen University Xiamen, Fujian China; 3 Department of Neurosurgery and Department of Neuroscience Fujian Key Laboratory of Brain Tumors Diagnosis and Precision Treatment, The First Affiliated Hospital of Xiamen University School of Medicine, Xiamen University Xiamen, Fujian China

**Keywords:** augmented reality, AR, shared decision-making, SDM, neurosurgery, preoperative communication, risk communication, informed consent

## Abstract

**Background:**

Preoperative risk communication is essential for shared decision-making (SDM) in neurosurgery; however, conveying complex neuroanatomy and surgical risks using traditional verbal explanations can limit understanding and contribute to dissatisfaction and medicolegal disputes. Augmented reality (AR) may provide patient-specific, interactive 3D visualization to support these conversations.

**Objective:**

This study evaluates whether AR-assisted preoperative risk communication improves objective understanding and other SDM-related outcomes, compared with communication supported by a conventional physical anatomical model within a standardized SEGUE-informed protocol.

**Methods:**

A prospective, single-center, randomized controlled trial was conducted with 62 neurosurgery communication recipients (patients when capable; otherwise, a legally authorized representative [LAR]). Patients were stratified by planned surgical approach (frontal, parietal, and occipital) and, within each stratum, were randomized into an experimental AR group and a control physical-model group. The primary outcome was postsession objective understanding, assessed by a multiple-choice knowledge questionnaire. Secondary outcomes were subjective understanding, communication satisfaction, pre-to-post anxiety changes, communication duration, and neurosurgeons’ communication skills from video recordings.

**Results:**

Of the 67 individuals screened, 62 communication recipients were enrolled and completed all assessments (patients, n=30; LARs, n=32; AR, n=32; and control, n=30). Objective understanding was higher with AR than with the physical model in the prespecified pooled comparison (*P*=.01). Communication satisfaction was also higher with AR (*P*<.001). There were no clear between-group differences in subjective understanding (*P*=.41), anxiety changes (ΔState-Trait Anxiety Inventory [ΔSTAI] Y-1, *P*=.37; ΔSTAI Y-2, *P*=.84), in-session face-to-face communication duration (excluding any presession AR technical preparation time; *P*=.73), or SEGUE scores (*P*=.60). Exploratory stratified analyses suggested larger comprehension gains with AR in the parietal and occipital approach strata.

**Conclusions:**

In a standardized preoperative SDM conversation, an integrated visualization-support package combining AR, patient-specific modeling, and interactivity improved neurosurgical decision makers’ objective understanding and satisfaction without prolonging in-session, face-to-face communication duration. Larger multicenter trials with longer-term outcomes are warranted to confirm effectiveness and to evaluate implementation and cost considerations.

**Trial Registration:**

ISRCTN Registry ISRCTN11483487; http://www.isrctn.com/ISRCTN11483487

## Introduction

Effective communication between health care professionals and patients has become a priority in modern medicine. Evidence indicates that high-quality communication improves patients’ comprehension and retention of medical information, enhances treatment adherence and coping skills, and ultimately benefits well-being, quality of life, and clinical outcomes [1,2]. More specifically, effective risk communication ensures that all parties share as accurate an understanding as possible of the factors relevant to clinical choices, thereby supporting shared responsibility for medical and surgical decisions [3]. In the context of neurosurgical decision-making, “risk communication” refers to a structured clinical process in which clinicians and decision makers exchange information about the expected benefits, probabilities, and consequences of potential harms, available alternatives, and remaining uncertainties, with the goal of supporting informed, values-concordant decisions. Risk communication therefore includes not only the content of the information shared but also how that information is organized and delivered (eg, plain language, opportunities for questions, and checking comprehension). This aligns with patients’ growing desire to be closely involved in their own care, reflected in the increasing use of shared decision-making (SDM) in surgical settings [4]. SDM is a joint process in which health care professionals and patients collaborate to choose among options [5,6]: clinicians present alternatives and explain risks and benefits; patients convey preferences and values; and, together, they grant or withhold consent to a specific intervention. Risk communication—the systematic exchange of information about the nature, magnitude, importance, and controllability of risks—is a core component of SDM [7]. When risk communication is effective, patients should understand the planned intervention and its risks, benefits, and reasonable alternatives [8].

In practice, preoperative conversations often encounter a dual challenge of providing “too little” and “too much” information. Oversimplified explanations are insufficient for sound decision-making, whereas unstructured, jargon-dense detail produces cognitive overload that impedes comprehension for nonexperts [9]. Studies consistently document suboptimal clinician-patient communication, leading to mutual misunderstanding [10]. In neurosurgical settings, time and workflow pressures can foster a checklist-style recital of risk factors. Although this approach may satisfy formal requirements, it weakens interactivity and relevance, limiting opportunities for patients and their legally authorized representatives (LARs) to discuss concerns openly and thereby diminishing the value of SDM [4,11].

As a specialty, neurosurgery manages a high volume of urgent and critical cases, with an overall complication rate near 14%, often resulting in severe functional disability [12]. When expectations of patients or LARs diverge from realistic prognoses, disputes and claims are more likely; indeed, neurosurgeons face the highest medicolegal risk among surgical disciplines [13]. Notably, inadequate or incomplete risk communication is among the most frequent contributors to litigation [14]. In time-pressured environments, a passive or compliance-based approach rarely enables patients to visualize and grasp risks linked to complex neuroanatomy, lesion margins, and eloquent functional areas [15].

To address these gaps, communication-support and decision-aid tools have been developed for diverse clinical contexts, including brochures, information leaflets, and mobile apps [16-19]. Augmented reality (AR) is increasingly leveraged across disciplines as a convergent, interdisciplinary technology. AR uses computer vision, graphics, and multimodal sensors to accurately register and superimpose virtual content (images, models, video, audio, and text) onto the user’s real-world view, creating a collaborative mixed environment for natural, real-time interaction and extended perception [20]. In medicine, AR is not new and has been applied across multiple specialties [21]. In neurosurgery, its capacity for real-time 3D reconstruction has supported preoperative planning, intraoperative navigation, and skills training [22,23]. However, comparative evidence on AR’s benefits for preoperative communication in neurosurgery remains limited. Clinicians hypothesize that AR’s intuitive 3D models can improve the transmission and understanding of complex information, but this requires systematic evaluation. In this study, “real-time support” refers to the in-session use of an AR-based, patient-specific 3D visualization as a shared reference during communication, enabling the clinician and the communication recipient to jointly orient to key structures, the target lesion, and clinically relevant high-risk regions as questions arise. Importantly, this term is used as a pragmatic description of an integrated visualization-support workflow (patient-specific anatomical content, head-mounted spatial presentation, and enhanced interactivity), rather than as a claim that the study isolates a single causal mechanism attributable to AR hardware alone.

Building on our institution’s experience with AR and incorporating Xvisio Technology’s spatial-perception interaction technology (Shanghai, China), we designed a study to evaluate AR-assisted preoperative risk communication in neurosurgery. In this study, we standardized the core informational items and communication structure across arms (SEGUE [*s*et the stage, *e*licit information, *g*ive information, *u*nderstand the patient’s perspective, and *e*nd the encounter]-informed checklist), and tested whether an AR-assisted, patient-specific 3D visualization—compared with a physical anatomical model—could improve decision makers’ understanding within the same risk-communication protocol. Using the SEGUE framework, we aim to implement AR within SDM in a standardized and streamlined manner. Our objectives are to (1) assess the effect of AR on comprehension and satisfaction; (2) develop an intuitive, reusable, and scalable workflow for preoperative SDM; and (3) provide a methodological basis for future multicenter, controlled studies. Accordingly, this proof-of-concept (PoC) trial is positioned as an exploratory, pragmatic comparison of 2 visualization-support packages (AR-assisted, patient-specific interactive 3D visualization vs a conventional, generic physical anatomical model), rather than a component-isolating test designed to separate spatial representation from interactivity or informational richness.

## Methods

### Trial Design

Our research was a prospective, single-center, randomized controlled PoC trial. According to the planned surgical approaches, participants were stratified into the frontal approach group (group 1), parietal approach group (group 2), and occipital approach group (Group 3), with randomization within each stratum to the experimental group (AR group) or the control group (physical-model group). Randomization within each surgical-approach stratum used a 1:1 allocation ratio (AR group vs physical-model group). The allocation sequence was generated using a computer-generated random-number list with simple randomization. To ensure allocation concealment, the sequence was implemented using a centralized web-based system prepared and maintained by an independent statistician. Baseline assessments (eg, State-Trait Anxiety Inventory [STAI] and Montreal Cognitive Assessment [MoCA]) were completed before group assignment. Participants were enrolled at the First Affiliated Hospital of Xiamen University, and assignment to interventions was implemented through the concealed allocation mechanism described above. Because of the nature of the interventions, clinicians and communication recipients were not blinded to group allocation. All recipient-reported outcomes (comprehension, anxiety, and satisfaction) were completed by the communication recipient. Neurosurgeons’ communication skills were evaluated using the SEGUE scale based on video recordings by independent assessors who were not involved in intervention delivery (assessors were not informed of allocation and scored recordings labeled only with study IDs).

This study aimed to preliminarily validate the feasibility and advantages of AR technology in preoperative risk communication for neurosurgery. By superimposing an individualized 3D virtual model from the AR headset onto the real environment and demonstrating it to the communication recipient, we compared this approach with the traditional physical model–assisted communication method across both subjective and objective dimensions. This comparison aimed to explore the impact of different communication-assistance technologies on recipients’ understanding of surgery-related issues (eg, surgical methods, surgical risks), communication satisfaction, and anxiety levels.

### Participants

This study included 67 patients who visited the Department of Neurosurgery at the First Affiliated Hospital of Xiamen University between January and September 2025 and were diagnosed with intracranial space-occupying lesions through auxiliary examinations after admission. As this is a communication intervention study, the enrolled participant was defined as the “communication recipient,” that is, the person who received the standardized preoperative risk communication and was primarily involved in SDM and consent for surgery. This was the patient when decisional capacity was intact; otherwise, an LAR served as the communication recipient and decision maker. Unless otherwise stated, all psychometric outcomes (STAI and MoCA) and postcommunication assessments (comprehension and satisfaction) refer to the communication recipient (patient or LAR).

All communication recipients (patients with decisional capacity or LARs, when applicable) provided written informed consent before enrollment. Participants were explicitly informed that the preoperative communication session would be video-recorded for research purposes (SEGUE-based assessment of communication quality), that refusal to be recorded would not affect clinical care, and that they could request discontinuation of recording and withdraw from the study at any time without penalty. Video recordings, questionnaire data, and study logs were deidentified by assigning unique study codes and stored on a password-protected, secure institutional drive with access restricted to the research team. Any patient imaging data used to generate the 3D models were processed in deidentified form for research use. Textboxes 1 and 2 detail the inclusion and exclusion criteria, respectively.

**Inclusion criteria.**

All the following inclusion criteria must be met:

**1. Study patients**

- Neurosurgery patients eligible for elective or semielective surgery with a feasible standard surgical approach.

**2. Technology**

- High-quality computed tomography or magnetic resonance imaging DICOM (Digital Imaging and Communications in Medicine) data from the hospital within 3 days before surgery. If needed, updated imaging must be completed before communication.

**3. Eligibility of the communication recipient**

- The recipient has no visual or auditory impairments, mental illness, or cognitive disorders. (1) The patient has the ability to provide informed consent and communicate and is therefore the primary communication recipient; or (2) the patient’s legally authorized representative is the primary participant in communication and decision-making when the patient lacks decisional capacity or has delegated authority. Decisional capacity was determined by the treating neurosurgeon based on routine clinical assessment and documented in the medical record.

**4. Age and language of the communication recipient**

- Aged 18-70 years; able to complete questionnaires and tests in Chinese (Mandarin); no gender restriction.

**5. Cooperation and compliance**

- Agreement to randomization, completion of on-site evaluation and follow-up before discharge, and willingness to wear augmented reality display devices when necessary (augmented reality group).

**6. Ethical consent**

- Signed informed consent for study participation (by the patient or legally authorized representative) and permission for the deidentified use of 3D imaging data for demonstration.

**Exclusion criteria.**

Any of the following conditions will result in exclusion:

**1. Clinical process–related**

- Emergency surgery patients for whom ≥24 hours is not available for preoperative assessment and communication.
- Patients with severe cognitive or consciousness disorders who are unable to complete the assessment and lack a qualified, legally authorized representative (eg, persistent delirium, severe aphasia, Glasgow Coma Scale score ≤12: E4+V3+M5 or E2+V5+M5).
- Difficulty cooperating with questionnaires and tests (eg, reading/writing disabilities, inability to complete the tests).
- Participation in similar intervention studies or communication-related intervention trials within the past 3 months that may affect primary outcomes (eg, understanding, decision conflict).

**2. Contraindications or high risks related to head-mounted augmented reality devices**

- History of photosensitive epilepsy or epilepsy clearly induced by visual stimuli.
- Vestibular dysfunction (eg, frequent dizziness attacks in the past 3 months or daily medication to control symptoms).
- Severe migraines with high sensitivity to bright light or dynamic images and frequent recent episodes.
- Severe visual impairments or eye diseases affecting augmented reality device use (eg, corrected vision ≤0.1, poor control of diplopia, postocular surgery recovery period).
- Severe hearing impairments with no equivalent alternative communication methods available on-site (eg, subtitles, hearing aids).
- Limitations on wearing and positioning: inability to safely wear head-mounted augmented reality devices due to external head fixation (eg, halo brace), large head dressings, or cervical fixation devices (eg, neck collar).
- Clear history of adverse reactions to augmented reality devices that led to discontinuation (eg, severe nausea, balance issues) and refusal to attempt use again.

**3. Others**

- Communication or ethical risks deemed inappropriate for inclusion (eg, family conflict affecting voluntariness).
- Legal restrictions or special populations requiring additional approval that has not been granted (eg, populations under compulsory supervision).

### Equipment

Head-mounted AR device (SeerLens B50R AR Glasses; Xvisio Technology) and a physical anatomical model (Hongyu Medical Education Equipment Co Ltd).

The AR intervention used the SeerLens head-mounted AR device. Key manufacturer-reported specifications relevant to clinical use (eg, display type, resolution, field of view, tracking method, weight, and computing/connection configuration) are summarized in Multimedia Appendix 1.

### Baseline Variables

At enrollment (before the communication session), we collected demographic characteristics of the communication recipient, including age, sex/gender, and educational attainment (recorded as the highest completed level and, for reporting in Table 1, collapsed into 3 categories to reflect the Mainland China compulsory education context and to reduce sparse subgroup cells). For LARs, we additionally recorded their relationship to the patient. When specified in the study protocol, baseline psychological and cognitive characteristics were also collected, including baseline anxiety assessed using STAI Y-1 and STAI Y-2 (before the communication session) and cognitive screening assessed using the MoCA (before the communication session). Baseline clinical and surgical variables (eg, diagnosis category and planned surgical approach) were extracted from the medical record.

**Table 1:** Summary of baseline characteristics of participants.^a^

Characteristics			Total (N=62)		Group 1 (N=20)				Group 2 (N=21)				Group 3 (N=21)				*P* value	
					Group 1A (n=9)		Group 1B (n=11)		Group 2A (n=11)		Group 2B (n=10)		Group 3A (n=12)		Group 3B (n=9)			
**Recipient type, n (%)**																	.46 (Fisher) (group 1A2A3A vs 1B2B3B)	
	Patient	30 (48)		4 (44)		5 (45)		7 (64)		4 (40)		6 (50)		4 (44)				
	Legally authorized representative	32 (52)		5 (56)		6 (55)		4 (36)		6 (60)		6 (50)		5 (56)				
**Gender,n (%)**																	.44 (Fisher) (group 1A2A3A vs 1B2B3B)	
	Male	35 (56)		5 (56)		6 (55)		8 (73)		5 (50)		7 (58)		4 (44)				
	Female	27 (44)		4 (44)		5 (45)		3 (27)		5 (50)		5 (42)		5 (56)				
**Education (highest educational attainment), n (%)**																	.77 (Fisher; group 1A2A3A vs 1B2B3B)	
	Middle school or below	19 (31)		2 (22)		3 (27)		4 (36)		3 (30)		4 (33)		3 (33)				
	High school	23 (37)		4 (44)		4 (36)		5 (45)		4 (40)		4 (33)		2 (22)				
	College or above	20 (32)		3 (33)		4 (36)		2 (18)		3 (30)		4 (33)		4 (44)				
**Age (years), mean (SD)**			54.48 (9.29)		57.89 (7.11)		57.45 (10.69)		50.45 (9.16)		54.5 (7.61)		54.58 (10.79)		52.22 (9.00)		.91 (group 1A vs 1B); .28 (group 2A vs 2B); .59 (group 3A vs 3B); and .74 (group 1A2A3A vs 1B2B3B)	
**STAI^b^** **-Form Y-Chinese version, mean (SD)**																		
	STAI Y-1	52.42 (9.41)		53.00 (10.17)		51.12 (10.62)		54.36 (8.86)		54.10 (8.97)		51.83 (11.24)		49.89 (7.08)		.70 (group 1A vs 1B); .95 (group 2A vs 2B); .63 (group 3A vs 3B); and .16 (group 1A2A3A vs 1B2B3B)		
	STAI Y-2	50.11 (9.11)		53.78 (8.20)		52.46 (8.74)		51.36 (9.33)		51.60 (9.16)		47.50 (9.89)		43.89 (7.18)		.73 (group 1A vs 1B); .95 (group 2A vs 2B); .34 (group 3A vs 3B); and .67 (group 1A2A3A vs 1B2B3B)		
MoCA^c^ score, mean (SD)			24.66 (3.88)		24.67 (3.28)		22.82 (4.67)		26.18 (2.68)		23.3 (5.68)		26 (3.5)^d^		25 (1)^d^		.31 (group 1A vs 1B); .17 (group 2A vs 2B); .29 (group 3A vs 3B); and .06 (group 1A2A3A vs 1B2B3B)	

^a^All baseline characteristics refer to the communication recipient (patient or LAR), unless otherwise stated. Groups 1A2A3A (n=32) included 17 (53%) patients and 15 (47%) LARs (patient-to-LAR 17:15), whereas groups 1B2B3B (n=30) included 13 (43%) patients and 17 (57%) LARs (patient-to-LAR 13:17). Recipient-type distribution did not differ significantly between arms (Fisher exact test *P*=.46).

^b^STAI: State Anxiety Inventory.

^c^MoCA: Montreal Cognitive Assessment (Chinese 7.1-Beijing).

^d^Reported as median (IQR) because the corresponding subgroup data were nonnormally distributed. The pooled comparison of objective understanding scores between the augmented reality and physical-model groups (groups 1A+2A+3A vs 1B+2B+3B) was prespecified as the single primary confirmatory analysis (2-sided α=.05). All other *P* values (stratum-specific comparisons and all secondary outcomes) are exploratory, unadjusted for multiple comparisons, and should be interpreted descriptively.

### Risk Communication Protocol and Standardization

#### Visualization Aid Intervention and Between-Arm Differentiation

The intended between-arm difference was the visualization aid used to support the same standardized explanation: the AR group received a patient-specific 3D visualization via a head-mounted display with adjustable views (eg, rotation/zoom and structure-visibility controls), whereas the control group received the conventional physical anatomical model used in routine practice. In both arms, recipients were encouraged to ask questions and interact with the tool jointly with the clinician.

#### Consistent Content

All sessions followed a prespecified, SEGUE-informed communication checklist to standardize the structure and core informational items delivered in both arms. The clinician explained (1) the diagnosis and relevant neuroanatomy; (2) the proposed surgical plan and procedural steps at a high level; (3) expected benefits; (4) major risks and high-risk regions, including the probability and potential consequences of key complications when such estimates were available; (5) alternatives (including conservative management, when applicable); and (6) postoperative expectations. Throughout the session, clinicians were instructed to minimize unnecessary jargon, respond to questions, and confirm understanding using standard comprehension checks (eg, asking the recipient to summarize key points in their own words).

#### AR Content Creation

A preconstructed digital 3D model was selected and adjusted according to each patient’s lesion location before use. Labels and approach- and risk-point annotations were overlaid, and the final model was preloaded and verified on the head-mounted AR device before the communication session.

#### Neurosurgeons Training (Before Communication)

##### Personnel Selection

Neurosurgeons were selected from the same institution with identical years of professional experience across all 3 communication levels; postcommunication assessment of neurosurgeons’ communication skills was conducted by an independent third-party researcher using the SEGUE scale.

##### Standardized Communication Training

This comprised the following: development and refinement of the risk-disclosure checklist, familiarization with head-mounted AR device operation, preparation of anticipated responses to questions, and avoidance of “overdisclosure” that may induce fear.

##### Operational Procedure Checklist

This considered the establishment of a standardized workflow (setup→alignment→demonstration→explanation→questioning→confirmation) to ensure consistency.

##### Situational Control

This involved the use of equally quiet and independent spaces, adherence to the same time window, and limitation of the number of onlookers.

##### Communication Time Limit

Communication duration was standardized across groups to minimize confounding from consultation length. Session duration was recorded, and any clinically necessary extensions were documented with justification. When required, additional routine counseling was provided after the study procedure to ensure adequate informed consent.

### Independent Design and Demonstration of Digital 3D Models

**Figure 1:**
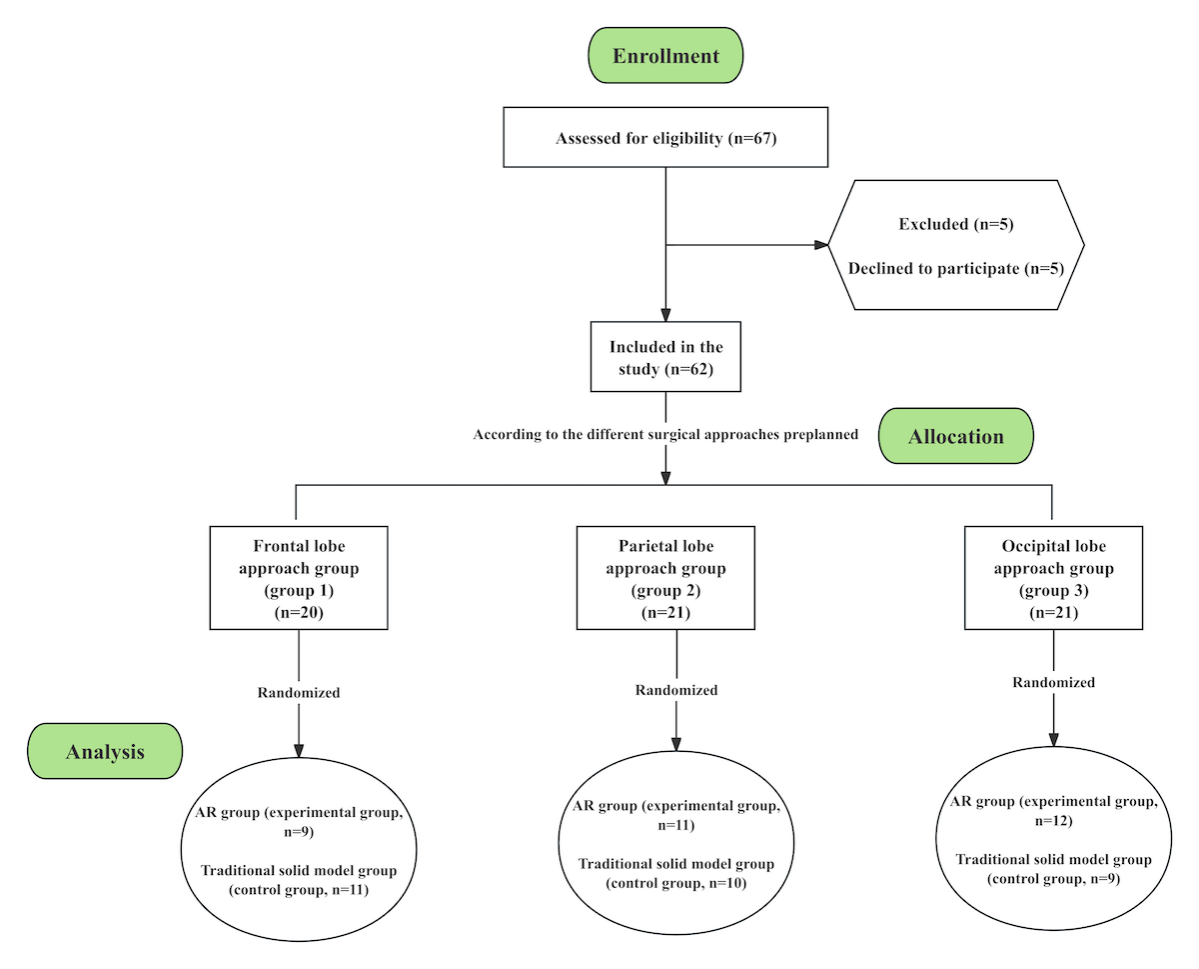
Study flowchart. AR: augmented reality.

Considering the characteristics of preoperative risk communication scenarios, this study used the open-source software 3D Slicer (The Slicer Community) to premodel classic lesion locations during digital 3D modeling. To protect patient privacy, deidentification procedures were implemented at the premodeling stage, including a comprehensive review of patient images to exclude any models exhibiting identifiable facial features. Visual inspection by 2 independent neurosurgeons (ZY and ZS) confirmed that none of the included prefabricated models were amenable to facial-feature recognition. As strict precision is not required for risk communication scenarios, the premade models were selected and adjusted according to the patient’s 2D imaging data before use (Figure 1A-C). Model adjustments can be made on a computer and quickly transferred to the SeerLens AR head-mounted device, maximizing time saved in preparation before communication.

During the model demonstration phase, the AR device used in this study (SeerLens B50R AR Glasses) was developed and manufactured by a domestic company (Multimedia Appendix 1). During the demonstration, the communication recipient uses the head-mounted AR device to superimpose a virtual projection onto the real patient’s head and, in combination with the neurosurgeon’s explanation, observes spatial information such as lesion size, depth, location, and adjacency. If necessary, the communication recipient can interact with the virtual model in real time through voice or gestures, under the neurosurgeon’s guidance, based on their needs (Figure 1D).

During AR-assisted sessions, the communication recipient viewed the patient-specific 3D anatomical model through a head-mounted AR display. The neurosurgeon did not wear a headset; instead, the neurosurgeon used a computer-based interface synchronized in real time with the recipient’s AR view. This workstation allowed the neurosurgeon to manipulate the model (eg, rotation, zoom, translation), adjust visual properties (eg, transparency/occlusion), and emphasize specific structures (eg, isolating the lesion and adjacent anatomy, highlighting/labeling), while monitoring the same model state shown to the recipient. In this way, the neurosurgeon could verify that the explanation referred to the correct virtual anatomical structures and direct attention through synchronized, on-model cues (Figure 1E).

### Interventions

Before randomization, eligible patients and their families were informed about the trial. Written informed consent was obtained from the patient when capable; otherwise, consent was provided by the patient’s LAR. During this process, 5 eligible patients declined participation. Ultimately, 62 communication recipients (patients or LARs) completed the Chinese version of the STAI-Form Y, which was used to evaluate state anxiety (State Anxiety Inventory, STAI Y-1) and trait anxiety (Trait Anxiety Inventory, STAI Y-2), with higher scores indicating higher levels of anxiety [24]. To evaluate the comprehension abilities of the communication recipients, cognitive function was assessed using the MoCA (Chinese 7.1-Beijing) [25]. Randomization was then performed within 3 strata. In the experimental group (AR group), communication recipients wore the SeerLens AR device when necessary, and the neurosurgeon superimposed the digital 3D model onto the patient’s real head, demonstrating the corresponding spatial structures based on the patient’s lesion location and explaining them to the communication recipient. Here, “real-time support” denotes the in-session use of the AR overlay as a shared spatial reference aligned to the patient’s lesion location, allowing the explanation to progress in step with the conversation and recipients’ questions. This wording describes the workflow and does not imply that the AR arm isolates a single mechanism (eg, spatial registration vs enhanced interactivity), nor that any observed effects can be attributed to AR hardware alone. In the control group (physical-model group), the neurosurgeon provided explanations with the assistance of a physical anatomical model. To minimize between-arm differences in informational content, clinicians followed a prespecified checklist of key information items derived from the SEGUE framework. The checklist covered (1) diagnosis and lesion location, (2) relevant neuroanatomical relationships, (3) the proposed treatment plan and surgical corridor/approach, (4) major risks and high-risk regions, (5) alternative options and the expected postoperative course, and (6) an opportunity for questions and teach-back (Multimedia Appendix 2). The checklist was identical for both arms; the only planned difference was the visualization tool used during explanation (AR-based patient-specific 3D model vs conventional physical model). Although informational items were standardized via the checklist, the AR aid inherently differs from the physical model in patient-specific anatomical specificity and software-enabled, dynamically adjustable visualization (eg, structure isolation/toggling and transparency adjustment); therefore, the between-arm contrast should be interpreted as a bundled comparison of these visualization-support features. In both arms, recipients were encouraged to ask questions and to interact with the tool jointly with the clinician.

After the communication session, the communication recipient completed postsession assessments, including (1) the Medical Knowledge Comprehension Questionnaire, which included a subjective understanding rating scale (Cronbach α=.88; Multimedia Appendix 3) to assess subjective comprehension and an objective understanding assessment (Multimedia Appendix 4): we used the Objective Assessment of Medical Knowledge Comprehension Following Risk Communication, a structured, directed questionnaire covering 4 domains (anatomical localization, treatment surgery, potential risks, and expected benefits), plus an open-ended item, developed to provide a pragmatic, objective quantification of recipients’ comprehension of the core information delivered during preoperative risk communication; (2) anxiety assessment (STAI-Form Y, Chinese version: STAI Y-1 and STAI Y-2); and (3) communication satisfaction assessment, using a self-developed rating scale (Cronbach α=.86; Multimedia Appendix 5). All questionnaires were self-administered by the communication recipient without assistance; no investigator administered the objective questionnaire or guided responses. Multiple-choice items were scored using a prespecified answer key.

Communication duration was measured in minutes by the neurosurgeon conducting the session and was defined as the face-to-face risk communication time from the start of the standardized explanation to the completion of the conversation. This metric did not include any presession technical preparation required for the AR workflow, which was completed before the communication session. The entire communication process was video-recorded so that independent researchers could later evaluate neurosurgeons’ communication skills using the SEGUE scale (Chinese version) [26,27].

### Outcomes

The primary outcome was objective understanding, assessed immediately after the communication session using the directed multiple-choice knowledge questionnaire (Multimedia Appendix 4). The key secondary outcome was subjective understanding, assessed using the subjective understanding rating scale (Multimedia Appendix 3). Other secondary outcomes were communication satisfaction (Multimedia Appendix 5), pre-to-post changes in anxiety (STAI Y-1 and STAI Y-2), communication duration, and neurosurgeons’ communication skills, assessed using the SEGUE scale based on video recordings.

### Sample Size

**Figure 2:**
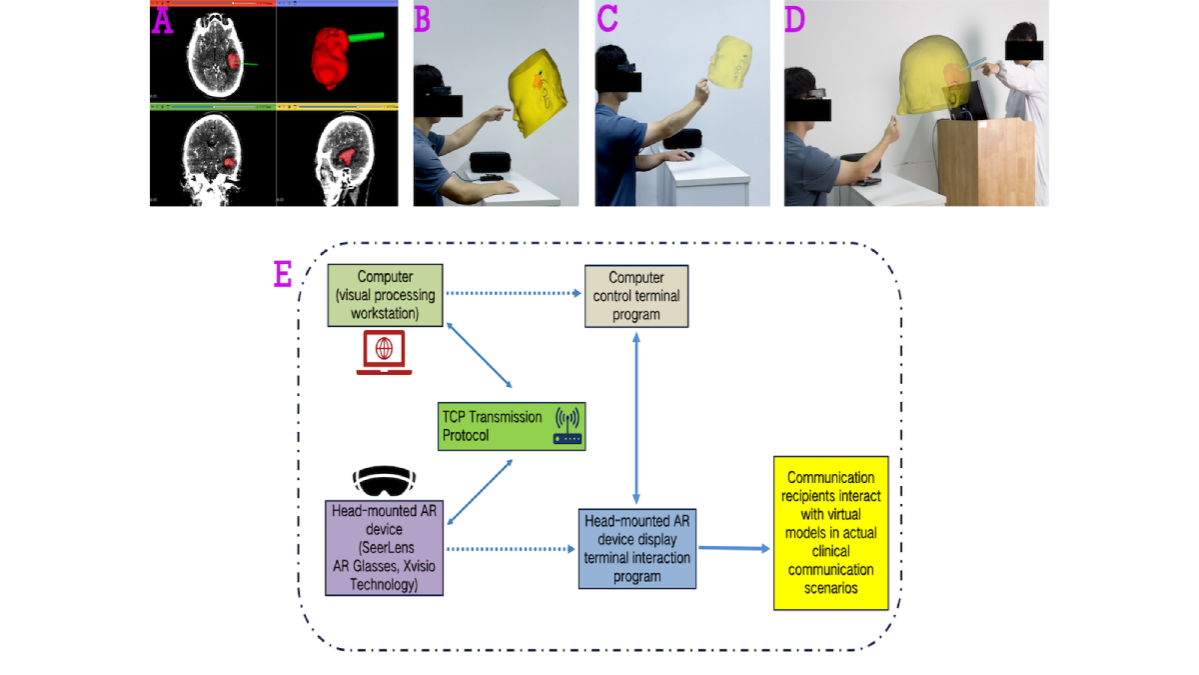
Schematic overview of the standardized clinician-recipient communication workflow under the SEGUE framework in routine clinical practice. (A) Digital 3D reconstruction derived from the patient's imaging data. (B and C) The communication recipient observes the virtual model from multiple perspectives according to their level of understanding and informational needs. (D) Illustration of the augmented reality (AR)–assisted communication approach in a clinical setting. The recipient views the virtual 3D model through a head-mounted AR device, while the physician does not wear a synchronized headset. Consequently, any physical pointing toward the recipient’s display is intended for approximate localization and illustrative purposes only, rather than precise identification of virtual structures. Visually aligned shared spatial reference and joint attention are achieved through a real-time synchronized computer interface, which allows the physician to manipulate the model and align explanations with the structures viewed by the recipient. (E) System architecture and data flow of the AR-assisted risk communication platform, comprising a synchronized computer control terminal and a head-mounted AR display terminal. TCP: terminal control platform.

We considered a 30% difference between the control and experimental groups to be clinically significant, based on the score range of the primary outcome measure (knowledge questionnaire scores). We assumed an expected effect size of *d*=1.50 (preset coefficient of variation=0.2), α=.05, and power (1–β)=0.85. The sample size was computed using G*Power (version 3.1.9.7). The required sample size was 10 participants per arm (20 total) for a single comparison. As we prespecified 3 anatomical strata and planned stratified analyses, we targeted approximately 20 participants per stratum (≈10 per arm), corresponding to a minimum total sample size of 60 across all strata (Figure 2A shows the per-stratum calculation). Although this sample size meets statistical requirements, as a single-center PoC study, it may still be constrained by the actual case volume. The primary analysis was conducted on the pooled cohort; powering per stratum was used as a conservative design choice to ensure adequate precision for the prespecified stratified analyses.

### Statistical Analysis

#### Data Summary Methods

Analyses were conducted in GraphPad Prism 9 (GraphPad Software). Continuous variables were summarized as mean (SD) when approximately normally distributed and as median with IQR (Q3-Q1) otherwise. Normality was assessed using the D’Agostino-Pearson test. Unless otherwise stated, tests were 2-sided. For between-group comparisons, Student *t* test was used when normality was not rejected and the Mann-Whitney *U* test otherwise.

#### Analytical Hierarchy and Multiple Comparisons

As this was a PoC study with multiple outcomes and planned stratum-specific comparisons, we prespecified an analytical hierarchy to reduce inflation of type I error from multiple comparisons.

#### Primary Confirmatory Analysis

The single primary confirmatory analysis (2-sided α=.05) was the pooled comparison of objective understanding scores between the AR and physical-model groups (groups 1A+2A+3A vs 1B+2B+3B).

#### Key Secondary Analyses (Exploratory)

Pooled comparisons of subjective understanding, communication satisfaction, anxiety changes (ΔSTAI Y-1 and ΔSTAI Y-2), communication duration, and SEGUE scores between the AR and physical-model groups were prespecified as exploratory secondary analyses. *P* values are reported without multiplicity adjustment and are interpreted descriptively as signals for future investigation rather than confirmatory evidence.

#### Stratified Exploratory Analyses

Within-stratum comparisons (1A vs 1B, 2A vs 2B, and 3A vs 3B) were conducted to explore potential heterogeneity by surgical approach. Given the small stratum-specific sample sizes, these analyses are hypothesis-generating only and should be interpreted with caution.

#### Recipient-Type Robustness Analyses

As the communication recipient could be either the patient or an LAR, we assessed whether recipient-type composition could confound communication outcomes. Recipient-type distribution (patient vs LAR), recipient sex, and education (highest educational attainment) were compared between arms using the Fisher exact test. We then conducted recipient-type robustness analyses for the primary and key secondary outcomes by reporting arm comparisons stratified by recipient type and by fitting linear models that included treatment group, recipient type, and a group × recipient-type interaction term. Sensitivity models adjusting for recipient type were used to evaluate whether the main findings were robust to recipient-type composition.

### Ethical Considerations

#### Study Approval

The study was approved by the Ethics Committee of the First Affiliated Hospital of Xiamen University, Xiamen, Fujian, China, in October 2024 (approval number 169). This trial was retrospectively registered with the ISRCTN registry (ISRCTN11483487; February 13, 2026). The first participant was enrolled in January 2025, and trial registration was completed on February 13, 2026. Registration was delayed due to the time required to complete and correct the registration materials. At study initiation, this investigator-initiated PoC risk-communication trial—embedded in routine preoperative SDM workflows and not involving any experimental clinical treatment—was mistakenly classified within our institutional governance process as a low-risk communication workflow/technology-feasibility study rather than as a registrable randomized clinical trial, despite the use of randomization. This was an administrative error and not an intentional omission.

To address potential reporting bias, the protocol originally reviewed and approved by the Ethics Committee in October 2024 (approval number 169) prespecified the eligibility criteria, randomization strategy, intervention procedures, primary outcome, key secondary outcomes, and analysis framework. A written statement issued by the Ethics Committee of the First Affiliated Hospital of Xiamen University, dated February 27, 2026, confirmed that the protocol registered in the ISRCTN registry remains consistent with the version originally reviewed and approved by the committee.

Written informed consent was obtained from all participants (patients or LARs), covering participation, the use of deidentified imaging data to generate 3D models for AR-assisted counseling, video recording of the consultation for SEGUE-based assessment, and completion of study questionnaires and assessments, in accordance with the Declaration of Helsinki (1964) and its subsequent amendments or comparable ethical standards.

#### Informed Consent

Written informed consent was obtained from all enrolled communication recipients before participation. When the communication recipient was an LAR, the LAR provided written consent; where feasible, patient assent was also sought. Participants were informed that participation was voluntary and that they could withdraw at any time without any impact on clinical care. The informed consent process covered completion of study questionnaires and the research use of relevant clinical information (eg, imaging data used to generate patient-specific 3D models and video recording of the communication session for SEGUE scoring).

#### Privacy and Confidentiality

All study data were deidentified and labeled using a unique study code. Any linkage file containing direct identifiers was stored separately from the analytic dataset and was accessible only to authorized study personnel. Data were analyzed and reported in aggregate to prevent the identification of individual participants.

#### Compensation

Participants did not receive any compensation for completing the study procedures.

#### Identifiable Information in Images/Multimedia Appendices

No images in the manuscript or multimedia appendices include identifiable information about individual participants (eg, faces, names, medical record numbers, or other direct identifiers). Photographs used to illustrate the AR-assisted communication workflow were taken with nonpatient volunteers (eg, with full-face blurring or cropping) and contain no patient-identifiable information.

### Patient or Public Contribution

Patients, caregivers, or members of the public did not serve as partners in the design, conduct, analysis, or reporting of this PoC randomized trial. Recruitment and communication occurred in time-sensitive preoperative settings, and the study focused on feasibility and measure refinement; therefore, establishing and supporting a Patient and Public Involvement and Engagement advisory group was not feasible at this stage.

## Results

### Baseline Characteristics

**Figure 3:**
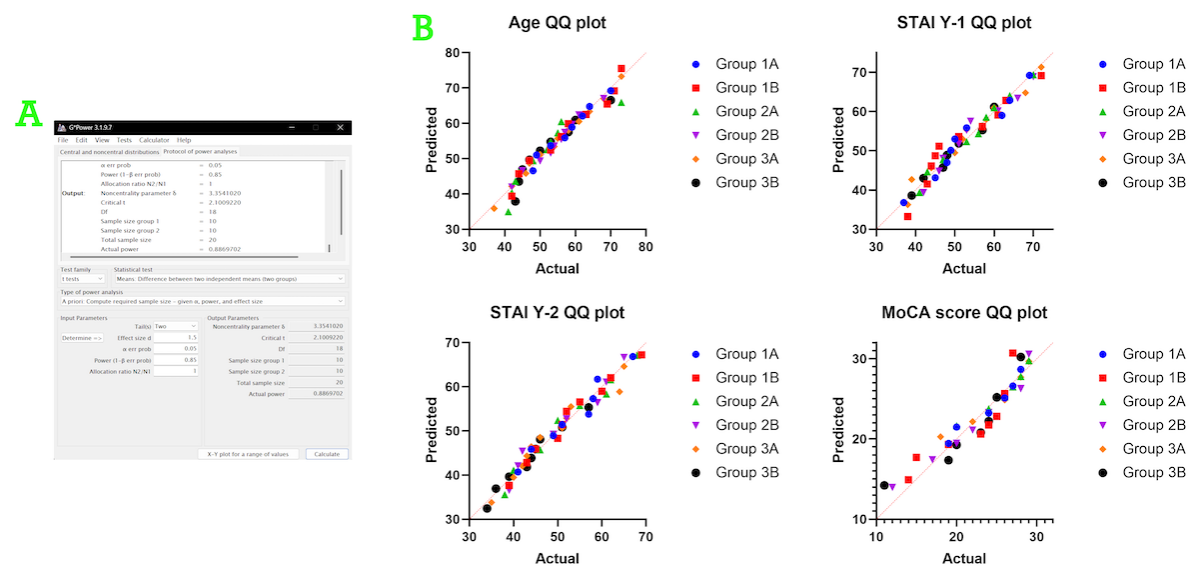
Sample size determination and statistical analysis preparation. (A) Results of the sample size calculation performed using G*Power (version 3.1.9.7). (B) Assessment of normality for baseline characteristics of all participants using the D'Agostino-Pearson test. MoCA: Montreal Cognitive Assessment; STAI: State-Trait Anxiety Inventory.

All 62 enrolled communication recipients completed the risk communication, with no dropouts. The primary communication recipient (who completed all study assessments) was the patient in 30 cases and an LAR in 32 cases (Table 1). Specifically, there were 20 participants in the frontal approach cohort (group 1), 21 in the parietal approach cohort (group 2), and 21 in the occipital approach cohort (group 3). Within the frontal approach group, the AR group (group 1A, experimental) comprised 9 patients, and the physical-model group (group 1B, control) comprised 11. Within the parietal approach group, the AR group (group 2A, experimental) included 11 patients, and the physical-model group (group 2B, control) included 10. Within the occipital approach group, the AR group (group 3A, experimental) included 12 patients, and the physical-model group (group 3B, control) included 9 (Figure 3 and see Multimedia Appendix 6 for the CONSORT [Consolidated Standards of Reporting Trials] checklist).

**Figure 4:**
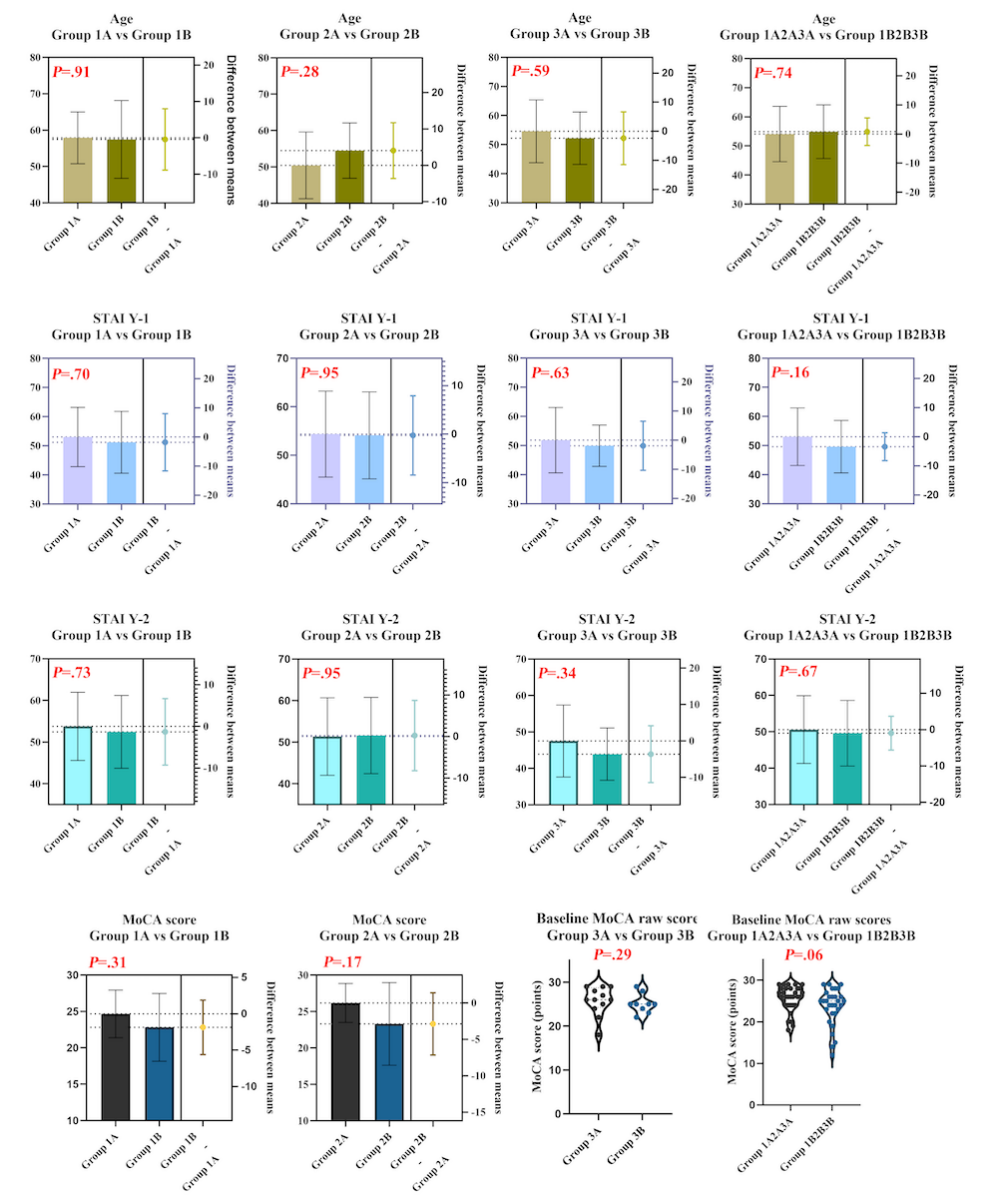
Visualization of participants' baseline characteristics. MoCA: Montreal Cognitive Assessment; STAI: State-Trait Anxiety Inventory.

After assessing normality (Figure 2B), we processed the data according to their underlying distributions. No baseline differences in demographics were observed among communication recipients across groups (Table 1). Before risk communication, there were no statistically significant pooled between-arm differences in baseline anxiety (STAI Y-1, *P*=.16; STAI Y-2, *P*=.67) or cognitive function (MoCA score, *P*=.06). Similarly, no statistically significant within-stratum baseline differences were observed (Table 1; Figure 4).

### Primary Outcome

#### Primary Confirmatory Analysis

**Figure 5:**
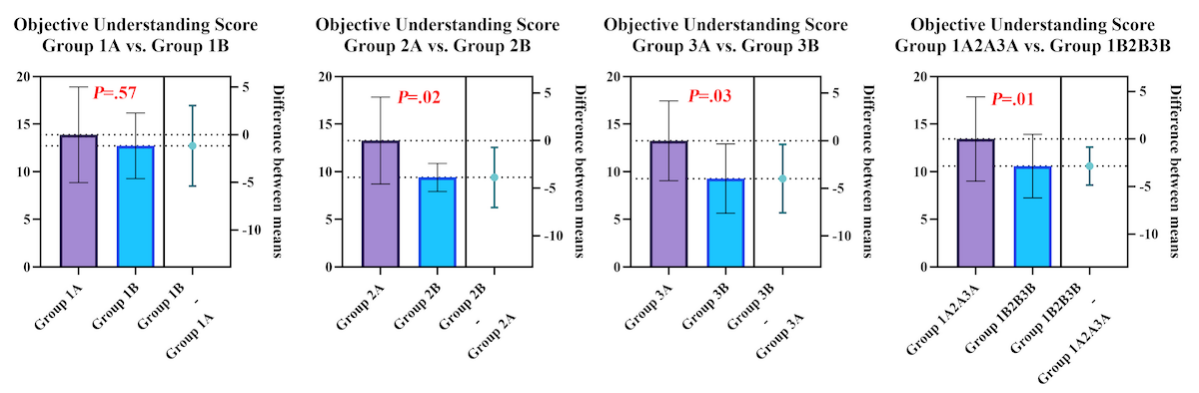
Visualization of participants' primary outcomes (objective comprehension scores).

The primary outcome (objective understanding) was measured using a 20-item multiple-choice questionnaire developed for this trial to operationalize the core informational elements delivered during the standardized, SEGUE-informed risk communication. Items were mapped to 4 predefined domains: anatomical localization (4 items), treatment/surgical plan (5 items), risks/complications (5 items), and expected benefits/expectations (6 items). Each item was scored 0-1 (correct/incorrect), with partial credit (0.5) awarded when a response reflected a partially correct understanding according to a prespecified rubric; the open-ended item was not included in the total score (range 0-20; Multimedia Appendix 4). In the prespecified pooled comparison, objective understanding scores were higher in the AR group than in the physical-model group (groups 1A+2A+3A vs 1B+2B+3B; *P*=.01; Table 2; Figure 5).

**Table 2:** The summary of research outcomes.^a^

Research outcomes		Total	Group 1 (n=20)			Group 2 (n=21)			Group 3 (n=21)			*P* values
			Group 1A (n=9)	Group 1B (n=11)	Group 2A (n=11)		Group 2B (n=10)	Group 3A (n=12)		Group 3B (n=9)		
Subjective understanding score, mean (SD) (range 8-40)			26.22 (6.28)	27.09 (4.68)	22 (17)^b^		21.00 (8.91)	24.50 (9.35)		21.78 (8.69)	.74 (group 1A vs 1B); .30 (group 2A vs 2B); .50 (group 3A vs 3B); and .41 (group 1A2A3A vs 1B2B3B)	
Objective understanding score, mean (SD)			13.89 (5.02)	12.73 (3.45)	13.28 (4.57)		9.40 (1.47)	13.25 (4.19)		9.28 (3.64)	.57 (group 1A vs 1B); .02^c^ (group 2A vs 2B); .03^c^ (group 3A vs. 3B); and .01^c^ (group 1A2A3A vs 1B2B3B)	
**STAI^d^** **-Form Y-Chinese version, mean (SD)**												
	STAI Y-1′	48.98 (10.08)	47.56 (9.85)	49.00 (13.32)	50.00 (9.02)		53.90 (10.45)	46.83 (9.56)		46.56 (7.65)	N/A^e^	
	STAI Y-2′	50.52 (9.09)	52.44 (10.26)	52.09 (9.44)	52.73 (7.95)		50.90 (9.76)	49.08 (9.31)		45.44 (7.81)	N/A	
	∆STAI Y-1	N/A	5.44 (7.35)	–3 (11)^b^	4.36 (8.86)		0.2 (13.52)	5.00 (19.32)		3.33 (10.11)	.37 (group 1A2A3A vs 1B2B3B)	
	∆STAI Y-2	N/A	1.33 (12.50)	0.36 (10.87)	–1.36 (9.83)		0.7 (9.50)	–1.58 (15.63)		–1.56 (11.68)	.84 (group 1A2A3A vs 1B2B3B)	
Communication satisfaction score (range 10-50)		N/A	40.67 (6.00)	32.18 (7.92)	35.91 (9.66)		24.40 (12.18)	36.17 (8.29)		27.78 (8.91)	.01^c^ (group 1A vs 1B); .03^c^ (group 2A vs 2B); .04^c^ (group 3A vs 3B); and <.001^c^ (group 1A2A3A vs 1B2B3B)	
Duration of communication (in-session) (minutes)		N/A	29.67 (7.87)	28.64 (6.79	28.27 (10.06)		27.90 (8.90)	26.67 (8.36)		25.11 (8.19)	.76 (group 1A vs 1B); .93 (group 2A vs 2B); .67 (group 3A vs 3B); and .73 (group 1A2A3A vs 1B2B3B)	
SEGUE^f^ score		N/A	22.22 (1.20)	21.91 (1.45)	21.64 (1.36)		20.10 (2.13)	20.75 (1.71)		21.67 (1.80)	.60 (group 1A vs 1B); .07 (group 2A vs 2B); .26 (group 3A vs 3B); and .60 (group 1A2A3A vs 1B2B3B)	

^a^All outcomes refer to the communication recipient (patient or legally authorized representative), unless otherwise stated.

^b^Reported as median (IQR) because the corresponding subgroup data were nonnormally distributed. The pooled comparison of objective understanding scores between the augmented reality and physical-model groups (groups 1A+2A+3A vs 1B+2B+3B) was prespecified as the single primary confirmatory analysis (2-sided α=.05). All other *P* values (stratum-specific comparisons and all secondary outcomes) are exploratory, unadjusted for multiple comparisons, and should be interpreted descriptively.

^c^*P* values indicate statistical significance.

^d^STAI: State Anxiety Inventory (STAI Y-1′ and STAI Y-2′ denote postsession scores). ΔSTAI (ΔSTAI Y-1 and ΔSTAI Y-2) was calculated as presession STAI minus postsession STAI (Δ=pre − post).

^e^N/A: not applicable.

^f^SEGUE*: s*et the stage, *e*licit information, *g*ive information, *u*nderstand the patient’s perspective, and *e*nd the encounter.

#### Stratified Exploratory Analyses

Within strata, objective understanding was higher in the AR group in the parietal approach stratum (group 2A vs 2B; *P*=.02) and in the occipital approach stratum (group 3A vs 3B; *P*=.03), but not in the frontal approach stratum (group 1A vs 1B; *P*=.57). These stratum-specific comparisons are underpowered and should be interpreted as hypothesis-generating (Table 2; Figure 5).

### Secondary Outcomes

#### Overview

All secondary outcome analyses were exploratory and were not adjusted for multiple comparisons; *P* values are interpreted descriptively.

#### Subjective Understanding (Exploratory)

**Figure 6:**
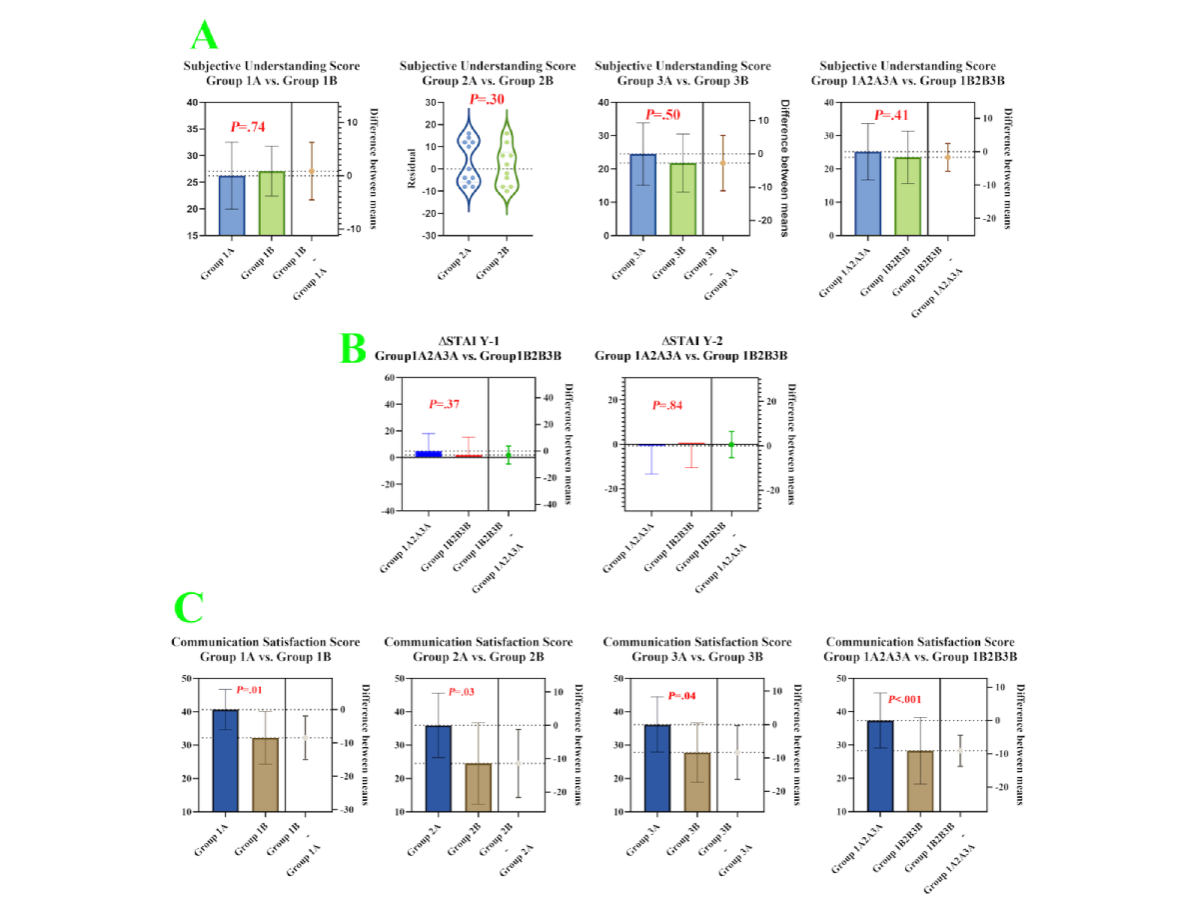
Visualization of secondary outcomes for all participants. (A) Subjective comprehension scores among communication recipients. (B) Changes in communication recipients' anxiety levels before and after the interaction. (C) Subjective satisfaction scores among communication recipients.

Subjective understanding scores did not differ between the AR and physical-model groups in the pooled comparison (*P*=.41) or within strata (*P*=.74 for group 1, *P*=.30 for group 2, and *P*=.50 for group 3; Table 2; Figure 6A).

#### Anxiety (Exploratory)

Notably, improvements in recipients’ anxiety did not differ meaningfully across communication modalities:

- STAI Y-1: for state anxiety, defined as momentary anxious reactivity, the pre-to-post change did not differ significantly between modalities (*P*=.37; groups 1A+2A+3A vs 1B+2B+3B).
- STAI Y-2: for trait anxiety, reflecting the general tendency to experience anxiety, the change from precommunication to postcommunication did not differ significantly (*P*=.84; groups 1A+2A+3A vs 1B+2B+3B; Table 2; Figure 6B).

#### Standardization-Related Outcomes

##### Communication Duration

Differences in communication time between groups were not statistically significant (*P*=.76: group 1A vs 1B; *P*=.93: group 2A vs 2B; *P*=.67: group 3A vs 3B; *P*=.73: groups 1A+2A+3A vs 1B+2B+3B).

##### SEGUE Score

There were no significant differences in the interviewing neurosurgeons’ communication skills across groups (*P*=.60: group 1A vs group 1B; *P*=.07: group 2A vs group 2B; *P*=.26: group 3A vs group 3B; *P*=.60: groups 1A+2A+3A vs groups 1B+2B+3B; Table 2).

#### Communication Satisfaction (Exploratory)

Regarding subjective satisfaction, scores were higher in the AR group across all strata (*P*=.01: group 1A vs group 1B; *P*=.03: group 2A vs group 2B; *P*=.04: group 3A vs group 3B). The AR group’s communication satisfaction scores were also higher than those of the physical-model group in the pooled comparison (*P*<.001: groups 1A+2A+3A vs groups 1B+2B+3B; Table 2; Figure 6C).

### Recipient-Type Distribution and Robustness Analyses

Recipient-type distribution was similar between groups (AR: 17/32, 53%, patients and 15/32, 47%, LARs; physical model: 13/30, 43%, patients and 17/30, 57%, LARs; Fisher exact test *P*=.46; Table 1). Recipient-type robustness analyses did not suggest that anxiety or satisfaction outcomes were driven by recipient-type composition. Interaction tests were not statistically significant for subjective understanding, satisfaction, or anxiety outcomes (*P*_interaction_=.22 for subjective understanding; *P*_interaction_=.07 for communication satisfaction; *P*_interaction_=.71 for ΔSTAI Y-1; and *P*_interaction_=.93 for ΔSTAI Y-2). However, for communication satisfaction, the group × recipient-type interaction approached conventional significance (*P*_interaction_=.07), with a substantially larger absolute AR – control difference among LARs (37.40 vs 24.71; Δ≈12.7 points) than among patients (37.29 vs 32.92; Δ≈4.4 points; Table 3).

Adjusting for recipient type did not change the overall conclusions (objective understanding *P*=.006). Robustness results are presented in Table 3.

**Table 3:** Recipient-type subgroup and sensitivity analyses (patients vs LAR^a^) for postcommunication outcomes.

Outcome	Patients’ AR^b^ (n=17), mean (SD)	Patients control (n=13), mean (SD)	*P* value (patients)	LARs’ AR (n=15), mean (SD)	LARs control (n=17), mean (SD)	*P* value (LARs)	*P* value interaction (group × recipient type)	*P* value adjusted
Objective understanding score	12.47 (5.03)	11.38 (2.97)	.47	14.53 (3.45)	9.97 (3.57)	<.001^c^	.09	.006^c^
Subjective understanding score	24.71 (8.51)	25.85 (7.14)	.69	25.73 (8.68)	21.65 (8.01)	.18	.22	.45
Communication satisfaction score	37.29 (7.65)	32.92 (8.63)	.16	37.40 (9.18)^d^	24.71 (9.77)^d^	<.001^c^	.07	<.001^c^
ΔSTAI^e^ Y-1	6.59 (15.12)	2.46 (13.06)	.43	3.00 (10.58)	1.41 (13.99)	.72	.71	.41
ΔSTAI Y-2	–1.06 (14.48)	–0.23 (10.34)	.86	–0.27 (10.67)	0.00 (10.69)	.94	.93	.86

^a^LAR: legally authorized representative.

^b^AR: augmented reality.

^c^*P* values show statistical significance.

^d^Reported as median (IQR) because the corresponding subgroup data were nonnormally distributed. *P* adjusted indicates the treatment-group effect from the sensitivity model adjusted for recipient type.

^e^STAI: State Anxiety Inventory.

## Discussion

### Principal Findings

In the prespecified pooled primary confirmatory analysis, AR-assisted preoperative risk communication in neurosurgery—compared with conventional aids centered on physical models—significantly improved recipients’ objective understanding of the current condition and surgery-related information. Although gains in self-perceived understanding were modest, recipients reported high subjective satisfaction with the communication. These effects are consistent with AR’s intuitive, 3D, and comprehensive spatial visualization. In addition, user-friendly head-mounted AR devices support real-time human-computer interaction, which may further enhance both subjective satisfaction with the process and objective understanding of the content. This pattern aligns with prior work showing that digital 3D planning tools improve the quality of surgeon-patient communication during informed consent and objectively promote patients’ understanding of their condition [4,19].

Notably, objective understanding did not differ significantly between groups in the frontal lobe approach subgroup (*P*=.57). This null finding should be interpreted cautiously. First, our a priori sample size calculation was based on the primary overall comparison and assumed a very large effect size (Cohen *d*=1.50). The frontal subgroup included only 9 and 11 participants and was therefore likely underpowered to detect moderate effects; the nonsignificant result may represent a type II error rather than evidence of no effect. Second, a clinically plausible explanation is that frontal approaches may involve relatively intuitive anatomical landmarks and a more straightforward surgical corridor; in such situations, a generic 3D physical anatomical model may already provide sufficient spatial cues for lay recipients, leaving limited room for additional improvement with AR (ie, a potential ceiling effect). By contrast, when lesions are deeper, risk-relevant structures are less visually accessible, or the planned corridor is more circuitous, AR may offer greater incremental value through multiangle exploration and dynamic, structure-specific highlighting and annotation. Taken together, the location/approach subgroup findings (including both significant and nonsignificant results) should be considered exploratory and hypothesis-generating.

Moreover, the control group demonstrated relatively low objective understanding scores (mean 9.40 and 9.28 out of 20 for the parietal and occipital regions, respectively), raising the question of whether physical model–based explanations are sufficiently effective for communicating complex surgical concepts. This low baseline performance may reflect the inherent challenges of explaining intricate surgical procedures using static models, or it may indicate that the standard physical model fails to adequately convey 3D anatomical relationships. This finding highlights the potential benefit of AR-assisted visualizations, which may better engage patients and provide more intuitive, spatially accurate representations.

Our study design could not fully disentangle the effect of personalization from that of the immersive technology. Specifically, the AR arm provided a patient-tailored 3D visualization based on the communication recipient’s routine imaging and adjusted to the lesion location, whereas the control arm used a generic physical anatomical model; therefore, the observed improvement in objective understanding may partly reflect content specificity rather than the AR delivery method itself. We did not include a patient-specific 2D imaging arm because this PoC trial was designed as a pragmatic study aligned with routine clinical practice in our mainland China setting, where generic physical anatomical models are among the most commonly used adjuncts for anatomy teaching and preoperative communication in many surgical departments. Moreover, in real preoperative neurosurgical risk communication, patient-specific 2D images alone are often difficult for nonexperts to interpret and may be insufficient to convey complex 3D relationships critical for risk discussion (eg, lesion depth, adjacency to eloquent regions, and surgical corridors). Finally, it is important to acknowledge that the use of AR in preoperative neurosurgical risk communication inherently combines 2 inseparable features—patient-specific personalization and an immersive, interactive experience. In real-world applications, these attributes do not operate in isolation but function as an integrated whole, jointly shaping communication outcomes. We therefore believe that the improved objective understanding of the surgical plan and related risks among communication recipients reflects the synergistic effects of personalization and immersive interaction. In addition, because head-mounted spatial presentation (in situ overlay) and enhanced interactivity (dynamic view manipulation) co-occurred in the AR workflow, this PoC design was not intended to determine which of these components primarily drove the observed differences.

We emphasize that AR’s 3D, immersive, and interactive features provide spatial anchors for apprehending complex intracranial anatomy and surgical pathways. In this study, “enhanced interactivity” refers to tool affordances that allow rapid switching between views (eg, rotation/zoom, structure toggling, transparency adjustment, and clipping planes) during joint clinician-recipient exploration of the 3D model; physical models also support interaction (eg, holding, rotating, and pointing), but with fewer view-manipulation affordances. In addition to conventional verbal explanations and physical anatomical models, AR head-mounted displays can provide an optional patient-specific 3D visualization. During the session, the clinician and the communication recipient can jointly orient to key structures, the target lesion, and clinically relevant risk regions using the same visual reference.

By rendering abstract spatial information visible and manipulable, AR affords an immersive, participatory experience that improves objective understanding and increases satisfaction. It is worth noting that although the mean changes in state anxiety (∆STAI Y-1) did not differ significantly between groups, the data revealed high variance across all strata (eg, SDs ranging from 7.35 to 19.32 in AR groups and reaching 13.52 in the parietal control group). This substantial variability suggests that high-fidelity visualization—whether via AR or physical models—may exert a “polarizing effect” on recipients’ emotional states. This interpretation is consistent with recent meta-analytic evidence indicating that, while virtual modalities consistently improve understanding, their impact on anxiety remains variable and nonsignificant compared with standard care [28]. For some individuals, the “unveiling” of the lesion’s precise location reduces the fear of the unknown, providing reassurance. Conversely, for others, the vivid and realistic depiction of the pathology may overwhelm their coping mechanisms. This aligns with modern psychological frameworks on “information avoidance,” which posit that detailed disclosure of threatening health information can paradoxically heighten distress in individuals prone to blunting coping styles, despite the objective gain in knowledge [29]. Therefore, the nonsignificant difference in mean anxiety scores likely reflects these opposing reactions canceling each other out. Meanwhile, self-rated understanding is strongly influenced by prior knowledge, expectations, and emotional state; over short intervals, it may not rise in parallel with objective measures, a finding consistent with our results [30]. These observations suggest that a single preoperative discussion is a necessary component of SDM but not a complete intervention chain. A comprehensive SDM process should integrate emotional support, psychoeducation, and expectation management into multi–time point, multichannel, continuous communication, coupled with counseling and follow-up across the preoperative-intraoperative-postoperative continuum. Within this chain, AR should function not merely as a display tool but as a vehicle for preference clarification and value prioritization. Integrating humanistic care with emerging assistive technologies is a likely future direction for clinician-patient communication.

Finally, there is another interesting result in this study. Our recipient-type robustness analyses also suggest a potentially meaningful trend in communication satisfaction. Although the group × recipient-type interaction did not reach the conventional threshold for statistical significance (*P*_interaction_=.07), the magnitude of the AR-associated improvement was substantially larger among LARs (≈12.7-point higher mean satisfaction vs control) than among patients (≈4.4-point higher vs control; Table 3). This pattern is hypothesis-generating, and the present PoC trial was not powered to test effect modification by recipient type; nevertheless, it is clinically plausible that surrogate decision makers may derive greater reassurance and perceived communication quality from patient-specific, manipulable visualization that clarifies anatomical localization and risk-relevant structures in real time. Future multicenter trials with adequate power should prespecify and evaluate recipient-type subgroup effects on satisfaction and related decisional outcomes.

### Comparison With Prior Work

During surgical consultations, clinician-patient dialogue often fails to establish a shared understanding of the anticipated care experience or alignment with patients’ overarching health goals [31,32]. Although surgeons must provide information to support autonomous decision-making, both surgeons and patients may treat informed consent as perfunctory. With its emphasis on 1-way information flow, the process affords limited real-time feedback from patients and often does not prompt reflection on the personal value of surgery or preparation for potential complications. Medicolegal reviews indicate that failure to explain surgical risks and adverse events is the most frequent allegation raised by plaintiffs [33]. SDM is designed to align therapeutic choices with patients’ objectives and, in principle, addresses this deficit [34]. Clinically, SDM commonly involves the neurosurgeon outlining to the patient or LAR the pros and cons of 2 or more therapeutic alternatives [35]. In this process, the mode of communication largely determines effectiveness; the judicious use of assistive technologies in preoperative risk communication can increase patient engagement in decision-making and strengthen both risk communication and SDM. At present, many artificial intelligence–designed decision-support tools (eg, decision aids and question-prompt lists) emphasize information analytics and behavioral prediction while paying relatively little attention to the bidirectional interactions needed to elicit, refine, and apply patient preferences or to support the communication process itself [36,37].

AR can help address these shortcomings by three-dimensionally visualizing personalized virtual models and overlaying them onto the physical clinical environment. Head-mounted AR devices allow real-time interaction via gestures or voice commands, enabling clinicians and patients to bridge the cognitive gap created by abstract spatial concepts. Under illness-related vulnerability and persistent information asymmetry, merely presenting options and describing their pros and cons does little to reduce information and power differentials—particularly in highly anxious contexts [38]. By overlaying virtual models onto the real-world setting, AR gives recipients access to otherwise latent anatomical relationships, substantially improving spatial understanding and the accuracy of risk localization. This, in turn, narrows—at least in the short term—the knowledge gap between clinicians and patients, as confirmed in our study.

Within the SEGUE framework, we minimized confounding from within-group variation in neurosurgeons’ communication competence and quantified skills using SEGUE scores to ensure appropriate assessment and control. This exploratory trial focused on the acceptability of AR assistance in real-world preoperative neurosurgical communication, the feasibility of implementation, and preliminary effects. The findings can guide industry partners in pursuing targeted product refinements for specific clinical scenarios and provide a practical foundation for improving models of neurosurgical preoperative risk communication.

From a clinical perspective, our results suggest that AR-assisted, patient-specific visualization may be a useful adjunct to standard preoperative risk and treatment plan communication, particularly when spatial understanding of neuroanatomy and surgery-related risk regions is critical. Importantly, AR should be viewed as a communication aid rather than a substitute for clinician communication skills; structured, empathic, and comprehensible explanations remain essential regardless of the visualization modality. At present, domestic head-mounted AR devices have not achieved full mass production, and versions for medical scenarios remain under continuous optimization. Critical components, including device reliability, hospital information system interfacing, data security, and legal scrutiny, are still evolving, which objectively limits short-term adoption. Nevertheless, with the maturation of the supply chain and software ecosystem, and with declining device costs, domestic AR devices are likely to be used more frequently in local health care contexts, enabling more targeted services and greater benefit for patients.

Implementation considerations such as usability, staff training burden, workflow integration, and privacy/data security compliance are important for real-world deployment; however, these factors (including costs and cost-effectiveness) were not evaluated in the present PoC trial. Therefore, our findings should not be interpreted as evidence that AR is ready for widespread adoption; rather, they motivate larger, multicenter, pragmatic studies, together with implementation and economic evaluations, to clarify feasibility and value across diverse health care settings.

### Limitations

Our study has several limitations. First, this was a single-center study with a modest sample size, which may limit generalizability. Multiple comparisons are an important limitation of this PoC study. We prespecified a single primary confirmatory comparison (pooled objective understanding) and treated all other comparisons as exploratory and unadjusted; nominally significant exploratory findings should be interpreted cautiously and confirmed in adequately powered trials with prespecified multiplicity-control strategies. Second, clinicians and participants could not be blinded to the visualization modality, introducing potential performance and novelty effects. Although SEGUE scoring was conducted by independent assessors who were not informed of group allocation, complete blinding may have been compromised because the AR headset could be visible in the video recordings; therefore, observer bias in SEGUE ratings cannot be excluded. Third, the costs of learning and process change deserve attention. Despite no significant difference in communication time between the AR and traditional model groups, widespread routine use of AR as a communication aid will require clinicians to spend time beforehand learning and adapting to the associated hardware and software processes. To maximize personalized and targeted communication effects, clinicians also need additional time for preparation before the conversation. We recognize that, for neurosurgical services with heavy workloads, such upfront commitments can create implementation challenges. Even so, additional time ought to be considered a normal element of precommunication preparation, because preoperative rehearsal is beneficial for surgeons, while its effect on care cadence should be balanced through improved processes and a clear division of labor. Furthermore, although we standardized the core informational items delivered in both arms, AR enables dynamic view manipulation and patient-specific visualization, which may alter the salience and perceived amount of information (ie, “effective information load”). Therefore, the observed improvement in objective understanding may reflect not only the technological modality itself but also the integrated features of an AR-assisted visualization package (patient-specific reconstruction and adjustable views). Future studies should include component-isolating comparators (eg, patient-specific 3D visualization delivered on a conventional 2D display, or generic 3D content delivered via AR with standardized interactivity levels) or factorial designs to disentangle personalization, spatial presentation, and interactivity. Fourth, participant heterogeneity should be noted. In keeping with real-world neurosurgical consent processes, some assessments were completed by LARs rather than patients. Although our intervention targets the communication recipient and preoperative decision maker, anxiety and comprehension may differ between patients and proxies. This PoC study was not powered to formally compare effects across recipient types.

### Conclusions

In this PoC randomized trial, AR-assisted preoperative risk communication was associated with higher objective understanding of surgery-related information and greater communication satisfaction than communication supported by a conventional physical anatomical model. As the AR arm inherently bundled patient-specific anatomical content, head-mounted spatial presentation, and dynamic view manipulation, these findings should be interpreted as evidence for an integrated visualization-support package rather than for AR hardware alone. AR’s interactive, 3D visualizations provide a more intuitive representation of complex neuroanatomical structures, improving spatial comprehension and enhancing communication satisfaction. While AR did not significantly reduce recipients’ anxiety compared with the physical model, it facilitated a more engaging and participatory experience, aligning with the principles of SDM. Within a standardized SEGUE-informed communication protocol, AR-assisted, real-time, patient-specific visualization may serve as a useful adjunct to SDM by improving recipients’ objective understanding of key anatomical and risk-related information.

## Appendix

Multimedia Appendix 1 SeerLens B50R AR Glasses (Xvisio Technology): a complete augmented reality solution for commercial use.

Multimedia Appendix 2 A prespecified, standardized risk communication checklist based on the SEGUE framework, applied by clinicians in both trial arms. SEGUE: set the stage, elicit information, give information, understand the patient’s perspective, and end the encounter.

Multimedia Appendix 3 Subjective Understanding Rating Scale (item structure and scoring system).

Multimedia Appendix 4 Objective assessment of medical knowledge comprehension following risk communication, comprising a 20-item questionnaire and scoring rubric.

Multimedia Appendix 5 Communication Satisfaction Rating Scale (item structure and scoring system).

Multimedia Appendix 6 CONSORT (Consolidated Standards of Reporting Trials) checklist.

## Abbreviations

AR: augmented reality
CONSORT: Consolidated Standards of Reporting Trials
DICOM: Digital Imaging and Communications in Medicine
LAR: legally authorized representative
MoCA: Montreal Cognitive Assessment
PoC: proof of concept
SDM: shared decision-making
SEGUE: set the stage, elicit information, give information, understand the patient’s perspective, and end the encounter
STAI: State-Trait Anxiety Inventory

## Data Availability

The datasets used or analyzed in this study are available from the corresponding author (ZW) upon reasonable request.
