# Supplementary material for: The Real-Time Support Role of Augmented Reality Technology in Shared Decision-Making in Neurosurgery Under the SEGUE Framework: Randomized Controlled Trial
Source: J Med Internet Res. 2026 Apr 17;28:e87198. doi: 10.2196/87198 (PMC13135152; doi:10.2196/87198)
Supplement: Multimedia Appendix 1 [file jmir_v28i1e87198_app1.pdf]

xvisio TECHNOLOGY

SeerLens™ B50R AR Glasses

Complete AR solution for commercial use

Package

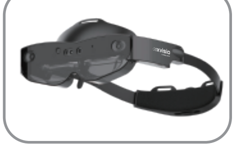

SeerLens™ B50R

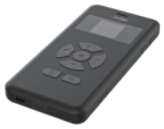

SeerPad™ One

SDK Software Development Kit

Xvisio AR Foundation

OpenVINO

AI Tool Chain

Optical Spec

Item	Spec	Remark
Optical Combiner	Birdbath	
Display Panel	1920x1080x2	Si-OLED, 3000nit
Frame Rate	60fps	
FOV	47°	Diagonal
IPD	63mm	
Eye Relief	20mm	
Contrast	500,000:1	
Transmittance	25%	

System Spec

Item	Spec	Remark
**Processor**	**Intel Movidius VPU**	
**Cameras | Sensors**		
-Stereo Mono Fish Eye	640x480, DFOV166°, 50 fps	2 camera system
-RGB	13M, DFOV79°, 30fps	
-IMU	9 Axis 1000Hz	
**SLAM Engine**	**1000Hz, 6DoF|3DoF**	**On board, Multiple modes**
**Depth Engine**	TOF HQVGA 30fps	**0.2-4m 1%**
**Audio Engine**	Stereo SPK| Mic	UAC API for Voice control
**Display Engine**	2D|3D mode, 1080P x2	Video over DP with USB Type C
**AI Engine**	On Board HW engine	OpenVINO model deployment
Power consumption	<5W	USB-C 2A Peak
Interface	USB Type C	DP output| USB input |Power
Weight |Size	<260g | 268Lx191Wx80H	LxWxH(mm)
**Computing Pack | OS**	**Rockchip 3588s|Seer AR OS**	**7.8V 4700mAh Battery**
SDK	Xvisio AR foundation on Unity	SLAM, Plane detection, Mapping

Innovating machine perception capability beyond human capacity

www.xvisiotech.com | +86 21 5290 0903 | contact@xvisiotech.com

xvisio
TECHNOLOGY

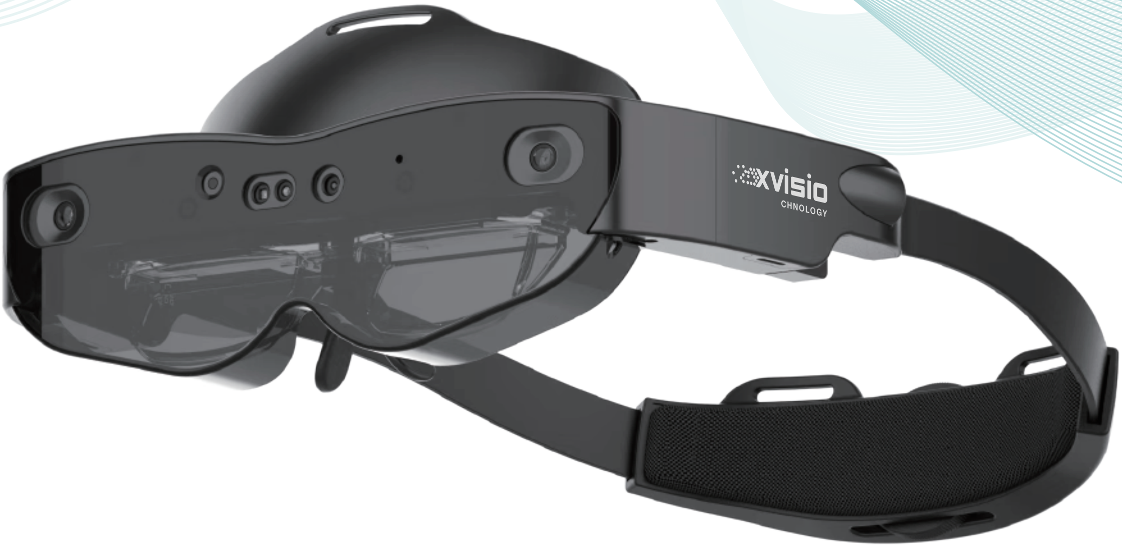

SeerLens™ B50R AR Glasses

Key Benefits

- Crystal clear FHD display with ultra-contrast for outdoor use
- Rugged and compact for both industrial and commercial use
- Plug and play for 5G mobile phone, laptop, computing pack
- **On board SLAM** for accurate head tracking
- **On board AI engine** can deploy 100+ models with one click
- **OpenVINO tool chain** to enable custom model training easily
- Low power and light weighted for long time operation
- Easy development environment with standard USB interface
- Feature rich Unity development kit for contents creation
- Forehead support for population with spectacles
- Intuitive 21 joint-point 3D hand gesture for easy control

Innovating machine perception capability beyond human capacity

www.xvisiotech.com | +86 21 5290 0903 | contact@xvisiotech.com
