# Supplementary material for: The Real-Time Support Role of Augmented Reality Technology in Shared Decision-Making in Neurosurgery Under the SEGUE Framework: Randomized Controlled Trial
Source: J Med Internet Res. 2026 Apr 17;28:e87198. doi: 10.2196/87198 (PMC13135152; doi:10.2196/87198)
Supplement: Multimedia Appendix 2 [file jmir_v28i1e87198_app2.docx]

**Multimedia Appendix 2** Standardized information checklist (same content items for both arms).

| Checklist item | Delivered in AR arm  (Y/N) | Delivered in control arm  (Y/N) |
| --- | --- | --- |
| 1. Confirm recipient identity/role (patient vs LAR) and establish agenda | [Y/N] | [Y/N] |
| 2. Explain diagnosis and lesion location (patient-specific imaging as applicable) | [Y/N] | [Y/N] |
| 3. Explain relevant anatomy and spatial relationships | [Y/N] | [Y/N] |
| 4. Explain proposed treatment plan / surgical corridor / key steps | [Y/N] | [Y/N] |
| 5. Explain major risks and high-risk regions (structured risk disclosure) | [Y/N] | [Y/N] |
| 6. Discuss alternatives and uncertainty | [Y/N] | [Y/N] |
| 7. Explain expected postoperative course / recovery | [Y/N] | [Y/N] |
| 8. Elicit questions and perform teach-back to assess understanding | [Y/N] | [Y/N] |

Y: Yes. N: No.
