# Supplementary material for: The Real-Time Support Role of Augmented Reality Technology in Shared Decision-Making in Neurosurgery Under the SEGUE Framework: Randomized Controlled Trial
Source: J Med Internet Res. 2026 Apr 17;28:e87198. doi: 10.2196/87198 (PMC13135152; doi:10.2196/87198)
Supplement: Multimedia Appendix 3 [file jmir_v28i1e87198_app3.docx]

A five-point Likert scale (I–V) was used for each item (scored 2, 4, 6, 8, and 10). The total Subjective Understanding score was calculated by summing the four items (range 8–40), with higher scores indicating greater subjective understanding by the communication recipient.

Cronbach's α=0.88

**Multimedia Appendix 3** Study-developed Communication Recipients Subjective Understanding Rating Scale

|  |  | **Level** | **Score** |
| --- | --- | --- | --- |
| Subjective Understanding of the Current Condition | After the physician’s explanation and communication, I do not understand my current condition at all. | I | 2 |
|  |  | Ⅱ | 4 |
|  | After the physician’s explanation and communication, I have a general understanding of my current condition, but it remains neither clear nor comprehensive. | Ⅲ | 6 |
|  |  | Ⅳ | 8 |
|  | After the physician’s explanation and communication, I have a clear and comprehensive understanding of my current condition. | Ⅴ | 10 |
| Subjective Understanding of the Current Treatment Plan | After the physician’s explanation and communication, I still do not understand the current treatment plan. | I | 2 |
|  |  | Ⅱ | 4 |
|  | After the physician’s explanation and communication, I have a general understanding of the current treatment plan, but I can only accept it passively. | Ⅲ | 6 |
|  |  | Ⅳ | 8 |
|  | After the physician’s explanation and communication, I have a clear understanding of the current treatment plan and can express my preferences/needs based on my understanding. | Ⅴ | 10 |
| Subjective Understanding of the Proposed Surgical Procedure and Its Risks | After the physician’s explanation and communication, I am not persuaded by the proposed surgical procedure and I do not understand the surgery-related risks. | I | 2 |
|  |  | Ⅱ | 4 |
|  | After the physician’s explanation and communication, I have a general understanding of the proposed surgical procedure, but it remains unclear; I am generally aware of the surgery-related risks, but my understanding is incomplete and I still have concerns. | Ⅲ | 6 |
|  |  | Ⅳ | 8 |
|  | After the physician’s explanation and communication, I am persuaded by the proposed surgical procedure and have a clear and comprehensive understanding of the surgery-related risks. | Ⅴ | 10 |
| Subjective Overall Comprehension of the Communicated Information | After the physician’s explanation and communication, I still do not understand the information communicated. | I | 2 |
|  |  | Ⅱ | 4 |
|  | After the physician’s explanation and communication, I have a general understanding of the information communicated, but I still have doubts/concerns. | Ⅲ | 6 |
|  |  | Ⅳ | 8 |
|  | After the physician’s explanation and communication, I have a clear and comprehensive understanding of the information communicated. | Ⅴ | 10 |
