# Supplementary material for: The Real-Time Support Role of Augmented Reality Technology in Shared Decision-Making in Neurosurgery Under the SEGUE Framework: Randomized Controlled Trial
Source: J Med Internet Res. 2026 Apr 17;28:e87198. doi: 10.2196/87198 (PMC13135152; doi:10.2196/87198)
Supplement: Multimedia Appendix 4 [file jmir_v28i1e87198_app4.docx]

### Objective Assessment Questionnaire of Medical Knowledge Comprehension Following Risk Communication

Questionnaire Instructions:

Dear patient or legally authorized representative,

Thank you for participating in this preoperative knowledge survey. This questionnaire is designed to assess your understanding—after discussion with your physician—of the patient’s current diagnosis, treatment plan, proposed surgical procedure, intraoperative risks, and potential complications. Through this survey, we aim to gauge your grasp of the medical information relevant to this admission and to further optimize the communication approaches we use before surgery. Your responses will be kept strictly confidential and used solely for medical quality improvement; they will not affect your treatment in any way. Please select the answers you believe are correct based on your recent conversation with the physician. Thank you for your cooperation.

Patient Basic Information:

Patient Name:__________ Name of Communication Recipient:__________ Relationship to Patient:__________

Date: __________(YYYY–MM–DD)

Ⅰ. Anatomical Localization

1. What is the patient’s current diagnosis?
   A. Brain tumor
   B. Cerebral aneurysm
   C. Cerebral thrombosis (ischemic stroke)
   D. Intracerebral hemorrhage
   E. Other, please specify: ________
2. On which side of the brain is the lesion located?
   A. Left
   B. Right
   C. Midline/central
   D. Uncertain
   E. Other, please specify: ________
3. Which eloquent functional area is the lesion close to?
   A. Motor cortex
   B. Language cortex
   C. Visual cortex
   D. Auditory cortex
   E. Other, please specify: ________
4. What is the main impact of the tumor at present?
   A. Compresses eloquent brain regions, causing limb weakness or language impairment
   B. Obstructs cerebrospinal fluid (CSF) circulation, leading to increased intracranial pressure (headache, vomiting)
   C. Triggers epilepsy (commonly referred to as “seizures”)
   D. I am not sure
   E. Other, please specify: ________

Ⅱ.Treatment Plan (Surgery)

1. What is the initial treatment plan we recommend?
   A. Primary surgical treatment, removing the lesion as much as possible
   B. Initiate radiotherapy and chemotherapy immediately, without surgery
   C. Endovascular intervention
   D. Pharmacotherapy
   E. Observation with periodic follow-up
   F. Other, please specify: ________
2. From which site does the physician plan to approach the lesion for removal?
   A. Via a forehead incision
   B. Via a posterior cranial (occipital) incision
   C. Via a transnasal approach
   D. Via a scalp incision
   E. Other, please specify: ________
3. Do you understand the main surgical pathway (corridor)?
   A. Through the frontal region, passing the cerebral cortex
   B. Entering from the back of the head, via the spinal cord
   C. Entering via the postauricular area into the ventricle
   D. Entering from the vertex (top of the head)
   E. Uncertain
   F. Other, please specify: ________
4. The surgical approach may be:
   A. Craniotomy
   B. Endoscopic surgery
   C. Minimally invasive surgery
   D. Uncertain
   E. Other, please specify: ________
5. In your view, the surgical pathway recommended by the physician is primarily related to:
   A. Lesion size
   B. Lesion location
   C. Patient age
   D. All of the above
   E. Other, please specify: ________

Ⅲ. Potential Risks

1. According to your understanding, which serious adverse events may occur during surgery?
   A. Massive intraoperative hemorrhage
   B. Major vascular injury (e.g., stroke)
   C. Injury to critical neurological functions (e.g., permanent motor, language, or visual deficits)
   D. All of the above
   E. Other, please specify: ________
2. Which common postoperative complications may occur?
   A. Wound infection or incision liquefaction
   B. Seizure(s)
   C. Intracerebral hemorrhage or cerebral edema
   D. Deep vein thrombosis (blood clot in the leg)
   E. All of the above
   F. Other, please specify: ________
3. If special circumstances are encountered during the operation, what is the surgical team’s primary principle?
   A. Achieve complete lesion removal at all costs
   B. Prioritize patient safety and preservation of critical neurological function; terminate the procedure if necessary
   C. Make real-time decisions based on the family’s opinions outside the operating room
   D. I am not sure
   E. Other, please specify: ________
4. Given the specific location of the patient’s lesion, which functional impact requires the most attention after surgery?
   A. Memory decline; personality or mood changes
   B. Language impairment (e.g., difficulty understanding or expressing speech)
   C. Contralateral limb weakness or numbness
   D. Visual field defect (difficulty seeing on one side)
   E. Balance dysfunction, unsteady gait
   F. I am not sure which specific risks are pertinent to me
5. How do you understand the risk–benefit trade-off of surgery?
   A. This is an absolutely safe operation with virtually no risk
   B. The surgery aims to address the current disease (e.g., reduce intracranial pressure, obtain pathology) but entails a range of known and potential risks that the team will endeavor to minimize
   C. The probability of adverse events is very high; the surgery is not worthwhile
   D. I have not formed a clear understanding of the trade-off

Ⅳ.Expected Benefits

1.What is the primary purpose of the surgery?
A. Complete removal of the lesion
B. Prevention of hemorrhage
C. Establishing a definitive diagnosis
D. All of the above
E. Other, please specify: ________

2.Which items are included in the surgeon’s expected goals for the operation?
A. Gross total resection of the tumor
B. Alleviation of the patient’s symptoms
C. Improvement in quality of life
D. All of the above
E. Other, please specify: ________

3.What do you think is the approximate likelihood of surgical success?
A. ≥90%
B. 70%–90%
C. 50%–70%
D. 30%–50%
E. Uncertain
F. After discussing with the physician, I believe the patient should not undergo surgery.

4.In your view, what are the main benefits the patient may gain from surgery?
A. Improvement in neurological function
B. Reduction of epileptic seizures
C. Enhanced quality of life
D. Complete tumor removal
E. Slowing tumor growth
F. Determination of tumor type (pathology)
G. All of the above

5.Approximately how long do you think recovery will take after surgery (to the condition you personally expect)?
A. Several days
B. Several weeks
C. Several months
D. More than six months
E. Cannot determine
F. Other, please specify: ________

6.Are you aware of possible post-operative adjuvant/ongoing treatments?
A. Yes; possibly radiotherapy or chemotherapy
B. Yes; possibly rehabilitation therapy
C. Uncertain
D. No

Do you have any other questions or concerns? Please feel free to write them below.

**Overall Score Grading (recommended for reporting/statistics)**

Excellent: ≥18 points (≥90%)

Good: 16–17 points (80–89%)

Pass: 13–15 points (65–79%)

Needs improvement: 10–12 points (50–64%)

Marked deficiency: ≤9 points (<50%)

Basic Scoring Rules (Total Score = 20 points)

Maximum per item = 1 point; an incorrect answer, selecting “Uncertain/I am not sure,” or leaving the item blank = 0 points.

Single-choice items: if multiple options are selected or erasures make the choice indeterminable, score = 0. If “Other, please specify” is selected and the written response is semantically equivalent to the answer key and accurate, award 1 point; otherwise 0.

Partial credit (0.5) was granted when responses reflected partially correct understanding according to a prespecified rubric; ambiguous cases were adjudicated by the research team.

Total score = sum across all 20 items (0–20 points).

Content validity was assessed by an expert panel (E=3) who independently rated item relevance on a 4-point scale (1=not relevant to 4=highly relevant); ratings ≥3 were considered content-valid. Item-level CVI values ranged from 0.33 to 1.00 based on three expert raters. The lowest I-CVI value (0.33) corresponded to the optional open-ended feedback item (“Do you have any other questions or concerns?”), which was intentionally retained to capture qualitative questions/concerns and was not included in the scored 20-item multiple-choice total (0–20)., and the scale-level average CVI (S-CVI/Ave) was 0.92.

Internal consistency reliability was evaluated using Cronbach’s alpha on the observed item scores (allowing partial credit), yielding α=0.80. To provide a dichotomous-test reliability estimate, we also calculated KR-20 after dichotomizing item scores (1=fully correct; 0/0.5=not fully correct), resulting in KR-20=0.81 (sensitivity analysis using ≥0.5). Item difficulty indices (proportion fully correct) ranged from 0.34 to 0.81, with no substantial floor/ceiling effects (observed total score range 1.5–19.5), supporting that lower scores likely reflect limited comprehension rather than uniformly excessive item difficulty.
