# Supplementary material for: The Real-Time Support Role of Augmented Reality Technology in Shared Decision-Making in Neurosurgery Under the SEGUE Framework: Randomized Controlled Trial
Source: J Med Internet Res. 2026 Apr 17;28:e87198. doi: 10.2196/87198 (PMC13135152; doi:10.2196/87198)
Supplement: Multimedia Appendix 5 [file jmir_v28i1e87198_app5.docx]

A five-point Likert scale (I–V) was used for each item (scored 2, 4, 6, 8, and 10). The total Communication Satisfaction score was calculated by summing the five items (range 10–50), with higher scores indicating greater subjective satisfaction with the communication on the part of the communication recipient.

Cronbach's α=0.86

Multimedia Appendix 5 Study-developed Communication Satisfaction Rating Scale

|  |  | **Level** | **Score** |
| --- | --- | --- | --- |
| Satisfaction with the Clarity of the Information Communicated | I find the information communicated abstract and difficult to understand, and I have difficulty accepting it. | I | 2 |
|  |  | Ⅱ | 4 |
|  | I find some parts clear, but other parts remain difficult to understand. | Ⅲ | 6 |
|  |  | Ⅳ | 8 |
|  | I consider the information communicated clear and unambiguous. | Ⅴ | 10 |
| Satisfaction with the Physician’s Communication Attitude | I consider the physician’s communication attitude negative and arrogant, lacking patience, and not taking the patient’s condition seriously. | I | 2 |
|  |  | Ⅱ | 4 |
|  | I consider the physician’s communication attitude generally good, but with shortcomings. | Ⅲ | 6 |
|  |  | Ⅳ | 8 |
|  | I consider the physician’s communication attitude positive, patient, and attentive to the patient’s condition. | Ⅴ | 10 |
| Satisfaction with Assistive Technologies Used in This Communication | I consider the assistive technologies to have provided no help in this communication. | I | 2 |
|  |  | Ⅱ | 4 |
|  | I consider the assistive technologies somewhat helpful, but their benefit was limited. | Ⅲ | 6 |
|  |  | Ⅳ | 8 |
|  | I consider the assistive technologies to have been very helpful in this communication. | Ⅴ | 10 |
| Satisfaction with My Emotional State During This Communication | I believe I was not treated with due respect during this communication, and my anxiety was not alleviated. | I | 2 |
|  |  | Ⅱ | 4 |
|  | I believe I received a certain degree of respect during this communication, and my anxiety was slightly alleviated. | Ⅲ | 6 |
|  |  | Ⅳ | 8 |
|  | I believe I received attention and respect during this communication, and my anxiety was alleviated. | Ⅴ | 10 |
| Overall Satisfaction | I am completely dissatisfied. | I | 2 |
|  |  | Ⅱ | 4 |
|  | I am generally satisfied, but there are still shortcomings. | Ⅲ | 6 |
|  |  | Ⅳ | 8 |
|  | I am very satisfied. | Ⅴ | 10 |
